# High Salt Intake Affects Visceral Adipose Tissue Homeostasis: Beneficial Effects of GLP-1 Agonists

**DOI:** 10.3390/biology14091171

**Published:** 2025-09-02

**Authors:** Vanessa Touceda, Leonardo Cacciagiú, Ignacio Barbani Moglie, Morena Wiszniewski, Valeria Sanchez, Romina C. De Lucca, Agustina Vidal, Paola Finocchietto, Silvia Friedman, Germán E. González, Verónica Miksztowicz

**Affiliations:** 1 Laboratorio de Patología Cardiovascular Experimental e Hipertensión Arterial, Instituto de Investigaciones Biomédicas (UCA-CONICET), Facultad de Medicina, Pontificia Universidad Católica Argentina, Buenos Aires C1107AFF, Argentina; toucedavanessa@gmail.com (V.T.); ignacioracedomoglie@campus.fmed.uba.ar (I.B.M.); germangonzalez@uca.edu.ar (G.E.G.); 2 Unidad de Investigación en Bioquímica Traslacional y Metabolismo (UBiTyM), Cátedra de Bioquímica General y Bucal, Facultad de Odontología, Universidad de Buenos Aires, Buenos Aires C1107AFF, Argentina; leonardo.cacciagiu@odontologia.uba.ar (L.C.); mwiszniewski@fmed.uba.ar (M.W.); pfinocchietto@gmail.com (P.F.); silvia.friedman@odontologia.uba.ar (S.F.); 3 Sección Bioquímica, Laboratorio Central, Hospital General de Agudos Teodoro Álvarez, Buenos Aires C1107AFF, Argentina; 4 Centro de Estudios Farmacológicos y Botánicos (CEFyBO), CONICET—Universidad de Buenos Aires, Buenos Aires C1107AFF, Argentina; 5 Instituto de Investigaciones Biomédicas (UCA-CONICET), Facultad de Medicina, Pontificia Universidad Católica Argentina, Buenos Aires C1107AFF, Argentina; valeriagsanchez@uca.edu.ar (V.S.); agustinavidal@gmail.com (A.V.); 6 Cátedra de Histología y Embriología, Facultad de Odontología, Universidad de Buenos Aires, Buenos Aires C1107AFF, Argentina; romina.delucca@odontologia.uba.ar; 7 Departamento de Medicina Interna, Facultad de Medicina, Universidad de Buenos Aires, Buenos Aires C1107AFF, Argentina; 8 CONICET-Consejo Nacional de Investigaciones Científicas y Técnicas, Buenos Aires C1107AFF, Argentina

**Keywords:** adipose tissue, high salt intake, vascular density, fibrosis, liraglutide

## Abstract

High salt (NaCl) intake has been associated with visceral adipose tissue (VAT) dysfunction independently of its impact on blood pressure. Liraglutide (LGT), a GLP-1 agonist, could be a potential therapeutic option. We investigated the impact of a chronic high-salt diet (HSD) on VAT homeostasis and evaluated the potential protective effects of LGT, a GLP-1 receptor agonist. Male C57BL/6 mice were fed a standard diet (Control, C) or 8% NaCl (HSD) for 15 weeks and subsequently treated with LGT or vehicle for 5 weeks. In VAT, histological characteristics, collagen deposition, vascular density, mitochondrial dynamics, oxidative stress, and adipokine expression were evaluated. The HSD significantly decreased body weight, VAT mass, and adipocyte size (*p* < 0.05). Moreover, it impaired vascular density and induced interstitial fibrosis (*p* < 0.01). LGT treatment improved vascularization and VEGF expression and reduced fibrosis (*p* < 0.05 vs. the HSD). The HSD induced oxidative stress and mitochondrial fragmentation, which were attenuated by LGT (*p* < 0.001). Leptin levels were elevated by the HSD (*p* < 0.05) and normalized with LGT, while adiponectin levels increased. In conclusion, excessive salt consumption induces structural and metabolic dysfunction in VAT. LGT therapy mitigates several of these adverse effects, supporting its potential as a novel strategy for managing salt-sensitive adipose tissue dysfunction.

## Simple Summary

Excessive dietary salt intake pervades Western diets due to the widespread use of processed foods and discretionary addition of table salt and is known to increase blood pressure, but it can also affect other tissues. In this study, we focused on visceral adipose tissue (VAT), which plays key roles in metabolism and inflammation. We investigated how a long-term high-salt diet (HSD) affects the structure and function of VAT in mice, and whether liraglutide (LGT), which is approved to treat diabetes mellitus and obesity, could offer protection. Our results showed that high salt intake led to reduced vascularity, increased fibrosis, adipokine imbalance, mitochondrial damage, and oxidative stress in VAT. However, treatment with LGT reversed many of these detrimental changes, improving tissue homeostasis. These findings suggest that excess salt intake can disrupt fat tissue health, even without weight gain, and that GLP-1-based therapies may prevent these effects. This research contributes to the understanding of how salt affects metabolism and highlights a potential treatment strategy to reduce the risk of related metabolic diseases in the general population.

## 1. Introduction

Excessive dietary intake of salt (NaCl) is one of the main causes of elevated blood pressure [1,2], cardiovascular disease (CVD) [3], and chronic kidney disease [4]. According to the World Health Organization, daily salt consumption should not exceed 5 g [5]; however, in Western countries, the average intake far surpasses this recommendation, mainly due to the high consumption of processed or industrialized foods and the addition of salt after cooking [6,7]. In recent years, there has been growing scientific interest in the impact of excessive salt consumption on tissue functionality. Current evidence suggests that high salt intake not only increases blood pressure but also has deleterious effects on different organs’ physiology [8]. It has been reported that high-salt diets (HSD) directly affect body weight and adipose tissue (AT) homeostasis; however, the results are limited and controversial, and many of the underlying pathophysiological mechanisms remain unclear [9,10,11].

Visceral AT (VAT) is an endocrine tissue capable of modulating the morphology and function of other organs. The maintenance of VAT homeostasis requires a tightly regulated remodeling process. Under physiological conditions, VAT plasticity and remodeling is associated with a balanced interplay between adipogenesis and angiogenesis [12]. Disruptions in the adipogenesis–angiogenesis balance undermine VAT remodeling, leading to dysregulated morphology and function. In recent years, numerous studies have highlighted the critical role of ATmitochondria in maintaining homeostasis [13]. Mitochondrial activity plays a pivotal role in regulating tissue dynamics, cellular functions, and intercellular communication; thus, maintaining proper mitochondrial dynamics is essential for adipocyte and tissue health.

Diets rich in carbohydrates and/or fats promote body weight gain and VAT expansion [14,15]. Expanded visceral fat is characterized by adipocyte hypertrophy and hyperplasia, fibrosis, impaired vascularization, oxidative stress, and reduced mitochondrial activity, hallmarks of VAT dysfunction [16,17,18]. However, the specific effects of chronic salt consumption on VAT homeostasis remain poorly understood. Research on the effect of high salt intake on VAT homeostasis has primarily focused on glucose uptake and insulin signaling [19,20], but whether chronic salt consumption affects VAT remodeling and function requires further investigation.

Given that VAT dysfunction is closely associated with different metabolic disorders, recent research has focused on identifying therapeutic strategies to modulate VAT function [21]. Glucagon-like peptide-1 (GLP-1) receptor agonists (GLP-1RAs) have emerged as promising pharmacological agents targeting VAT, and liraglutide (LGT) is one of the most studied [21,22]. Given the evidence that LGT reduces body weight and VAT mass, it has been approved by the U.S. Food and Drug Administration (FDA) for the treatment of type 2 diabetes mellitus and obesity [23,24]. GLP1-R is a member of the G protein-coupled receptor family found on the surface of many cell types, including pancreatic b-cells, neurons of the central nervous system, adipocytes, vascular smooth muscle cells, and endothelial cells, among others [25]. When activated by a ligand, it typically initiates the production of cyclic adenosine monophosphate and the consequent activation of its downstream effectors [26]. It has been previously reported that the in vitro activation of GLP-1R by GLP-1 [27] or LGT [28] promotes the differentiation of 3T3-L1 preadipocytes through the ERK1/2 and Hippo-YAP signaling pathways, respectively. Nevertheless, several mechanisms underlying the actions of GLP-1RAs on VAT remain unclear, and additional signaling cascades are currently under investigation [25].

Our aim was to investigate the impact of chronic high salt intake on VAT homeostasis and to evaluate the potential beneficial effects of LGT treatment. To achieve this goal, we evaluated histological characteristics, extracellular matrix collagen content, vascular density, oxidative stress, mitochondrial dynamics, and adipocyte function markers in VAT from an animal model fed an HSD and treated with LGT.

## 2. Materials and Methods

### 2.1. Animal Model and Experimental Design

Male C57BL6/J mice were maintained under controlled-temperature (20–22 °C), humidity, and air flow conditions with a fixed 12-h light/dark cycle. At 8 weeks of age, animals were randomly divided into two groups and fed for 20 weeks as follows: Control group (C, *n* = 24) fed standard chow and high-salt diet group (HSD, *n* = 21) fed a diet containing 8% NaCl. Animals had free access to food and drinking water. After 15 weeks, each group was subdivided according to the subcutaneous administration of LGT (200 μg/kg/day) or a saline solution (equivalent volume) for 5 weeks: C (*n* = 13), C + LGT (*n* = 11), HSD (*n* = 10), and HSD + LGT (*n* = 11). The dose and duration of LGT treatment were selected based on previously published studies [18,29,30]. Body weight, food, and water consumption were registered weekly and caloric intake was calculated throughout the experimental period. Food efficiency ratio (FER) was calculated for each diet as FER (%) = (weight gain (g)/food intake (g)) × 100.

All the procedures were carried out in our Bioresources facilities at the Biomedical Research Institute (BIOMED, UCA-CONICET) of the Pontificia Universidad Católica Argentina. The experimental protocol N° 004/2022 was approved by the Institutional Committee for the Care and Use of Laboratory Animals (CICUAL) of BIOMED in line with the NIH’s Guide for the Care and Use of Laboratory Animals [31].

### 2.2. Systolic Blood Pressure

Systolic blood pressure (SBP, mmHg) was evaluated at the beginning of the experiment (basal), before LGT treatment (at week 15), and at the end of the experiment (at week 20). Animals were trained for one week before the measurement of SBP by plethysmography according to the tail cuff method [32]. SBP values were registered for three consecutive days each time in a quiet environment and avoiding stressful conditions for the animals. The results of the three measurements were averaged and a single SBP value was considered for each animal.

### 2.3. Sample Collection

#### 2.3.1. Urine

To collect urine samples, mice were individually housed in metabolic cages for 48 h under the same conditions described previously. The initial 24-hour period was considered as an acclimatization phase and the collected urine was discarded. Total urine volume from the following 24 h was collected and then centrifuged at 950× *g* for 10 min. The resulting supernatant was stored at −20 °C for further determination of renal function biomarkers.

#### 2.3.2. Serum

At the end of the experimental period, following a 4-h fasting period, animals were euthanized by the intraperitoneal administration of a sodium pentobarbital overdose (i.p, 150 mg/kg). Blood samples were collected and centrifuged at 950× *g* for 10 min; serum was obtained and stored at −20 °C for subsequent analyses of sodium, creatinine, glucose, and lipid profile levels.

#### 2.3.3. Tissues

Epididymal adipose tissue (EAT), used as a representative depot of visceral fat, was excised, weighed, fractionated, and stored in liquid nitrogen for subsequent molecular analyses; one fraction of the tissue was fixed with 4% buffered formalin (pH 7.0) for 48 h at 4 °C prior to tissue processing. Another fraction was immediately fixed with a solution containing 4% paraformaldehyde, 2% glutaraldehyde, and 5% sucrose in phosphate-buffered saline for assessments of mitochondrial morphology by transmission electron microscopy (TEM).

Tibias were also removed to measure their length, and both the final body weight and EAT mass were normalized to the tibia length.

### 2.4. Biochemical Determinations

In serum, glucose, total cholesterol (t-chol), HDL-chol, and triglyceride (TG) levels were determined by enzymatic colorimetric methods in a Cobas-501 autoanalyzer (Roche Diagnostics, Mannheim, Germany). The non-HDL chol level was calculated as the difference between t-chol and HDL-chol levels as representative of atherogenic lipoprotein levels.

To evaluate renal function, urine and plasma sodium and creatinine levels (uNa, plNa, uCr, and plCr) were evaluated by indirect Ion-Selective Electrode and kinetic colorimetric methods, respectively. Diuresis was measured and standardized creatinine clearance (CrCl) and the fractional excretion of sodium (FENa) were calculated according to the following formulas:

(1) $$Standardized\;CrCl=\frac{uCr}{plCr}\times D/1440$$

Standardized CrCl—standardized creatinine clearance (µL/min);

uCr—urinary creatinine (mg/dL);

plCr—plasma creatinine (mg/dL);

D—diuresis (µL of urine/24 h);

1440—conversion factor (min/24 h).

(2) $$FENa=\frac{uNa\times plCr}{plNa\times uCr}\times 100$$

FENa—fractional excretion of sodium (%);

uNa—urinary sodium (mmol/L);

plNa—plasma sodium (mmol/L);

uCr—urinary creatinine (mg/dL);

plCr—plasma creatinine (mg/dL).

### 2.5. Histological Analysis

For histological analysis, fixed EAT samples were dehydrated in a graded ethanol series and embedded in paraffin. Slices of 5 μm thickness were obtained using a Histocore Multicut microtome (Leica) and stained with hematoxylin–eosin reagents to quantify the adipocyte size (µm^2^) and number (number of adipocytes/mm^2^), as previously described [18]. Diameters of adipocytes were determined to estimate the cell volume according to the following formula [33]:

(3) $$CV=\frac{\pi }{6}\times ({3S}^{2}\;\times \;{CD}^{2})\times CD$$

CV = cellular volume (pL);

S^2^ = variance of CD;

CD = cellular diameter.

Histological characteristics were evaluated by a single blinded operator. Quantification was performed by light microscopy at 400× magnification, and 20 randomly selected high-power fields from different regions of the same tissue section per animal were analyzed. Images were analyzed using Image ProPlus software (Version 6.0, Media Cybernetics Corp).

### 2.6. Adipose Tissue Collagen Content

The interstitial collagen content in EAT was assessed in paraffin-embedded sections stained with Picrosirius Red. Collagen deposition was quantified as the percentage of collagen area relative to the total tissue area. All image analyses were performed blind to group allocation. Analysis was conducted under a light microscope at 400× magnification, and 10 randomly selected high-power fields from different regions of the same tissue section per animal were evaluated. Images were evaluated by a single blinded operator using Image Pro Plus software (Version 6.0, Media Cybernetic Corp).

In addition, the total collagen content in EAT was determined by quantifying hydroxyproline [34]. Briefly, a fraction of EAT was dehydrated and hydrolyzed in 6 N hydrochloric acid at 100 °C overnight. The residual acid was evaporated using a vacuum centrifuge at 35 °C for 4 h, and the resulting residue was suspended in distilled water. Samples and standards (commercial hydroxyproline standard, Cat #H5534, Merck KGaA, Darmstadt, Germany) were oxidized with chloramine-T solution and subsequently reacted with Ehrlich’s reagent. The resulting chromophore was measured at 550 nm using a spectrophotometer.

### 2.7. Vascular Density in EAT

Vascular density in EAT was evaluated by a fluorescence assay. Paraffin-embedded slices were dewaxed with xylene and rehydrated with ethanol. Slices were then incubated with a neuraminidase solution (3.3 U/mL, Cat. N#2876, Merck KGaA, Darmstadt, Germany) for 1 h at room temperature. Then, sections were incubated for 2 h at room temperature with rhodamine-labeled Griffonia simplicifolia lectin (0.12 mg/mL, Cat. #RL-1102, Vector Laboratories, Newark, NJ, USA) to label cell membranes and fluorescein-labeled peanut agglutinin lectin (0.01 mg/mL, Cat. #FL-1071, Vector Laboratories, Newark, NJ, USA) to visualize the interstitial matrix. Nuclei were stained with DAPI (Vectashield^®^ Cat. #H-1200-10, Vector Laboratories, Newark, NJ, USA). Representative images were acquired using a confocal microscope (Zeiss LSM 980, Zeiss, Oberkochen, Germany), and blood vessels were quantified using Image Pro Plus software (Version 6.0, Media Cybernetics Corp). The blood vessel density was normalized to the adipocyte number.

Immunohistochemistry was performed to evaluate Vascular Endothelial Growth Factor (VEGF) expression as a marker of angiogenesis. EAT sections were processed simultaneously under identical conditions using the streptavidin–biotin peroxidase method. Slides were incubated overnight in a humidified chamber with a mouse monoclonal anti-VEGF antibody (Cat. #sc-7269, Santa Cruz, Dallas, TX, USA). Immunoreactivity was revealed with 3,3′-diaminobenzidine (DAB) and counterstained with hematoxylin–eosin. Negative controls were included by omitting the primary antibody. To quantify VEGF levels, five non-overlapping high-power fields (400× magnification) were randomly selected for each sample. The DAB-positive area was identified, and the percentage of positive staining was calculated relative to the total tissue area. The average percentage of VEGF-positive areas per field was used for statistical analysis.

### 2.8. Oxidative Stress Evaluation

#### 2.8.1. Lipid Peroxidation

Lipid peroxide levels in EAT were determined by measuring thiobarbituric acid-reactive substances (TBARS). Briefly, tissue samples were homogenized in 15 mmol/L KH_2_PO_4_/K_2_PO_4_, 60 mmol/L KCl (pH 7.4). Homogenates were centrifuged at 1000× *g* for 10 min at 4 °C, and 50 µL of the resulting supernatant was mixed with 25 µL of 10% SDS and 465 µL of a thiobarbituric acid solution (8 mg/mL in 10% acetic acid, pH 3.5). The mixture was incubated at 95 °C for 1 h. Fluorescence was then measured using a fluorometer with excitation at 515 nm (λₑₓ) and emission at 555 nm (λₑₘ).

#### 2.8.2. Antioxidant Enzyme Activity

Catalase (CAT) and superoxide dismutase (SOD) activities were assessed using colorimetric assays. Ten milligrams of EAT were homogenized in 500 µL of 50 mM sodium phosphate buffer (NaH_2_PO_4_/Na_2_HPO_4_, pH 7.4) containing a protease inhibitor cocktail (P8340-1ML, Merck KGaA, Darmstadt, Germany). Homogenates were centrifuged at 9500× *g* for 10 min at 4 °C, and the supernatants were collected for enzymatic analysis. CAT activity was determined by monitoring the decrease in absorbance at 240 nm due to hydrogen peroxide decomposition. SOD activity was measured based on the inhibition of 1,2,3-trihydroxybenzene autooxidation at 420 nm.

### 2.9. Mitochondrial Morphology Evaluation

TEM was used to analyze mitochondrial morphology in EAT samples. Tissue fragments (~1 mm^2^) were fixed with a solution containing 4% paraformaldehyde, 2% glutaraldehyde, and 5% sucrose in phosphate-buffered saline, followed by post-fixation with 1% osmium tetroxide for 2 h and an incubation with 1% uranyl acetate in 50% ethanol for 1 h. Samples were then washed with 50% ethanol, dehydrated through a graded ethanol series, cleared with acetone, and embedded in Vestopal resin. Grids were prepared and stained with uranyl acetate and lead citrate. Imaging was performed at 100 kV using a Zeiss EM-109-T TEM (Zeiss, Oberkochen, Germany). Mitochondrial morphology was evaluated in at least 8–10 randomly selected fields per animal, analyzing 3–4 mitochondria per field, at magnifications ranging from 3000× to 30,000×. Mitochondria were classified as tubular if their length exceeded three times their width, and as fragmented if they exhibited a rounded shape. All image analyses were performed blind to the group allocation.

### 2.10. Hypoxia-Inducible Factor-1α (HIF-1α), Uncoupling Protein-1 (UCP-1), Leptin, and Adiponectin Expression

HIF-1α, UCP-1, leptin, and adiponectin expression levels were assessed by quantitative real-time PCR (RT-qPCR). Total RNA was extracted from 100 mg of VAT using Quickzol^®^ reagent (0102, Kalium Technologies, Buenos Aires, Argentina), following the manufacturer’s instructions. Reverse transcription was carried out using M-MLV reverse transcriptase (M1701, Promega, Madison, WI, USA) to obtain complementary DNA. Amplifications by qPCR were performed using a QuantStudio^TM^ 1 Real-Time PCR Instrument (Applied Biosystems by Thermo Fisher Scientific, Waltham, MA, USA) with Taq DNA polymerase (5 U/µL, Cat# EA0101, PB-L, Buenos Aires, Argentina). Gene expression was normalized to β-actin as a reference gene. Relative expression levels were calculated using the comparative threshold cycle (Ct) method (2^−ΔΔCt^), as all primer pairs showed similar amplification efficiencies, according to previously described protocols [35].

β-actin: Forward GGCTGTATTCCCCTCCATCG/Reverse CCAGTTGGTAACAATGCCATGT.

HIF-1α: Forward GATCTCGGCGAAGCA/Reverse ACCATGTCGCCGTCATC.

UCP-1: Forward CAGTACCCAAGCGTACCAAG/Reverse GTTCCAGGACCCGAGTC.

Leptin: Forward GAGACCCCTGTGTCGGTTC/Reverse ATGAAGTCCAAGCCAGTGACC.

Adiponectin: Forward CAGTGGATCTGACGACACCAA/Reverse CGTCATCTTCGGCATGACTG.

### 2.11. Statistical Analysis

Data are presented as means ± standard deviations (SDs) or medians (ranges) according to a normal or skewed distribution, respectively. Group comparisons were analyzed using unpaired Student’s *t* test, the Mann–Whitney U test, ANOVA or Kruskal–Wallis test, and Bonferroni’s or Dunn’s post hoc test according to the group number and data distribution. Pearson’s or Spearman’s analyses of parametric or non-parametric variables were used to determine correlations between parameters. Prior to statistical analyses, Grubbs’ and ROUT tests were applied to identify potential outliers. GraphPad Prism 8 software (Version 8.0.1, GraphPad) was used for statistical analysis and for graphical design. A *p*-value < 0.05 was considered statistically significant.

## 3. Results

### 3.1. General Characteristics of the Animal Model

As shown in Table 1, the initial body weight and SBP were similar between groups. At week 15, the HSD group presented a lower body weight (*p* < 0.001) and higher SBP (*p* < 0.001) than the C group (Table 1). Food and water intake were higher in the HSD group (*p* < 0.01 and *p* < 0.001, respectively), without differences in caloric intake between groups (Table 1). At the end of the protocol, there was no difference in tibia length between groups, indicating normal growth. The HSD and HSD + LGT groups presented lower body weight than the C group (*p* < 0.001), even after correction for tibia length (*p* < 0.001) (Table 2). Caloric intake and food consumption were similar between groups, and water intake continued to be higher in the HSD and HSD + LGT groups (*p* < 0.001 and *p* < 0.05, respectively, vs. the C group). FER of the HSD and HSD + LGT groups was lower than the C group (*p* < 0.05). SBP remained higher in the HSD group in comparison with the C group (*p* < 0.01), and it significantly decreased in the HSD + LGT group (*p* < 0.05 vs. the HSD group).

The HSD and HSD + LGT groups presented a significant decrease in EAT mass in comparison with the C group (*p* < 0.001), even after tibia length correction (*p* < 0.001) (Table 2).

### 3.2. Circulating and Renal Functional Parameters

In Table 3, circulating parameters are shown. No differences were observed in serum sodium, creatinine, glucose, and lipid levels between groups. Diuresis was increased in the HSD and HSD + LGT groups (*p* < 0.001 and *p* < 0.05 vs. the C group, respectively), as well as uNa (*p* < 0.001 and *p* < 0.05 vs. the C group, respectively) and FENa (*p* < 0.001 vs. the C group) (Table 4). The HSD group presented a higher standardized CrCl (*p* < 0.05 vs. the C group), which decreased in the HSD + LGT group (*p* < 0.05 vs. the HSD group) (Table 4).

### 3.3. Histological Analysis of EAT

**Figure 1:**
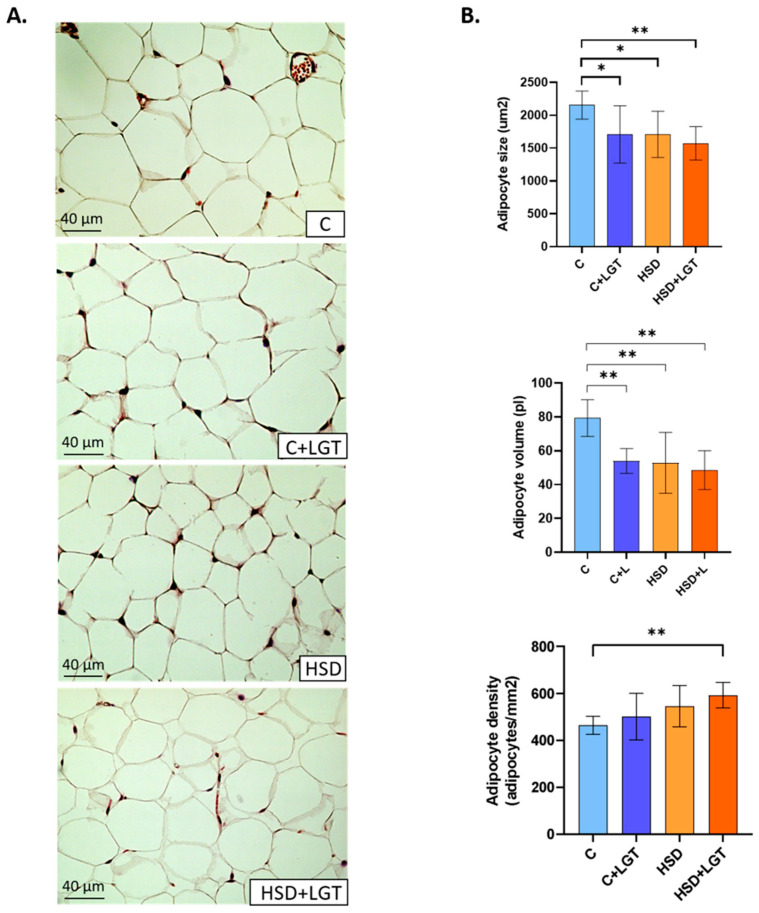
Histological characteristics of EAT. (**A**) Representative images of H-E-stained sections; (**B**) histological characteristics of EAT in the Control (C), Control + LGT (C + LGT), HSD, and HSD + LGT groups. Data are presented as means ± SDs. One-way ANOVA followed by Bonferroni’s post hoc test. * *p* < 0.05, ** *p* < 0.01.

The general histological characteristics of EAT were evaluated using hematoxylin–eosin staining (Figure 1). The HSD and HSD + LGT groups presented smaller adipocytes than the C group, as indicated by a significant decrease in the cell area (*p* < 0.05 and *p* < 0.01, respectively) and volume (*p* < 0.01). Adipocyte density was higher in the HSD + LGT group compared to the Control group (*p* < 0.01).

### 3.4. Adipose Tissue Collagen Content

**Figure 2:**
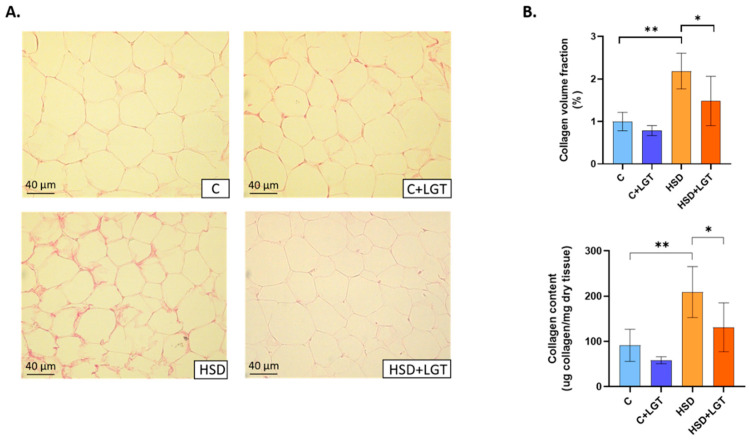
Interstitial collagen content in visceral adipose tissue. (**A**) Representative images of Picrosirius Red-stained sections. (**B**) Collagen volume fraction (%) quantification and total collagen content measured through the hydroxyproline assay in the Control (C), Control + LGT (C + LGT), HSD, and HSD + LGT groups. Data are presented as means ± SDs. One-way ANOVA followed by Bonferroni’s post hoc test. * *p* < 0.05, ** *p* < 0.01.

Fibrosis in EAT was evaluated by histological and colorimetric methods. Picrosirius Red staining revealed that the HSD markedly increased interstitial collagen fraction (*p* < 0.01), which the hydroxyproline assay confirmed. Significantly, LGT treatment reversed this fibrosis (*p* < 0.05) (Figure 2).

### 3.5. Vascular Density in EAT

**Figure 3:**
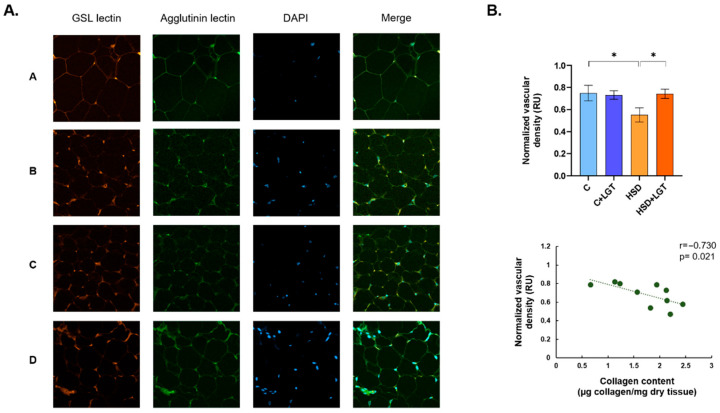
Vascular density measured by a fluorescence assay. (**A**) Representative images of rhodamine-labeled GSL lectin, fluorescein–peanut agglutinin lectin and DAPI–stained sections of representative EAT samples from the Control (A), Control + LGT (B), HSD (C), and HSD + LGT (D) groups. (**B**) Quantification of vascular density and its correlation with the total collagen content (Spearman’s test, *n* = 10). Data are presented as means ± SDs. One-way ANOVA followed by Bonferroni’s post hoc test. * *p* < 0.05.

To expand the study of the EAT characteristics under HSD conditions, vascular density was evaluated by a fluorescence assay (Figure 3). The HSD group presented a significant decrease in vascular density in comparison to the C group (*p* < 0.05), while it significantly increased in the HSD + LGT group compared to the HSD group (*p* < 0.05). Finally, the vascular density was inversely associated with the collagen content (r = −0.730, *p* = 0.021).

**Figure 4:**
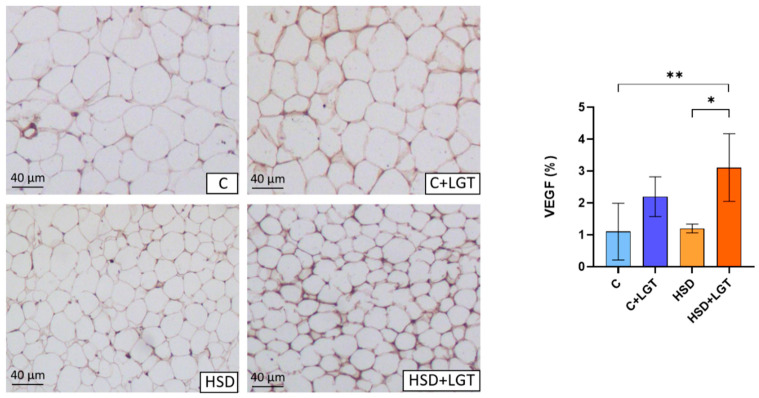
Immunohistochemical analysis of Vascular Endothelial Growth Factor expression. Representative images of EAT from the Control (C), Control + LGT (C + LGT), HSD, and HSD + LGT groups. VEGF levels are expressed as percentages (%) of the total tissue area. Data are presented as means ± SDs. One-way ANOVA followed by Bonferroni’s post hoc test. * *p* < 0.05, ** *p* < 0.01.

VEGF was assessed by immunohistochemistry as a marker of angiogenesis. VEGF expression was significantly increased in the HSD + LGT group compared to the HSD (*p* < 0.05) and C (*p* < 0.01) groups (Figure 4).

### 3.6. HIF-1α Expression

**Figure 5:**
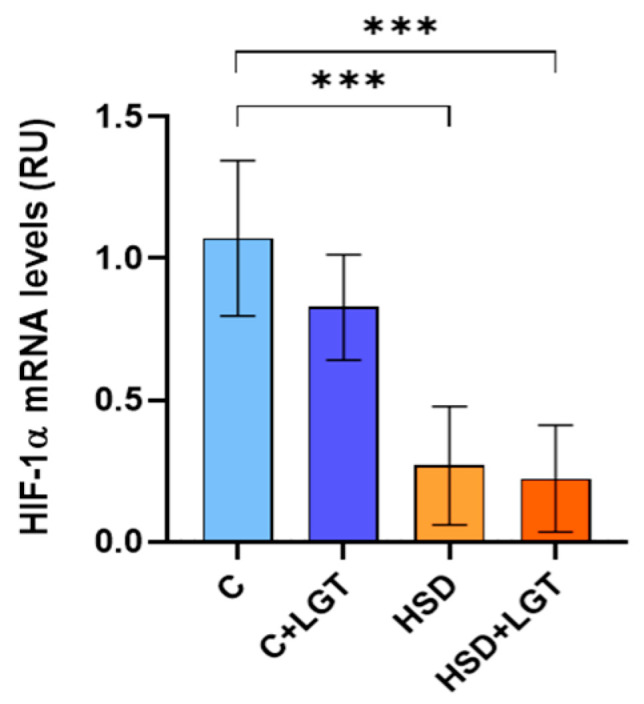
HIF-1α expression. HIF-1a mRNA levels in epididymal adipose tissue (EAT) from the Control (C), Control + LGT (C + LGT), HSD, and HSD + LGT groups. Data are presented as means ± SDs. One-way ANOVA followed by Bonferroni’s post hoc test. *** *p* < 0.001.

Given the observed reduction in vascular density induced by salt consumption, HIF-1α mRNA levels were quantified by RT-qPCR. HIF-1α expression was significantly reduced in the HSD and HSD + LGT groups compared to the C group (*p* < 0.001) (Figure 5).

### 3.7. Oxidative Stress

**Figure 6:**
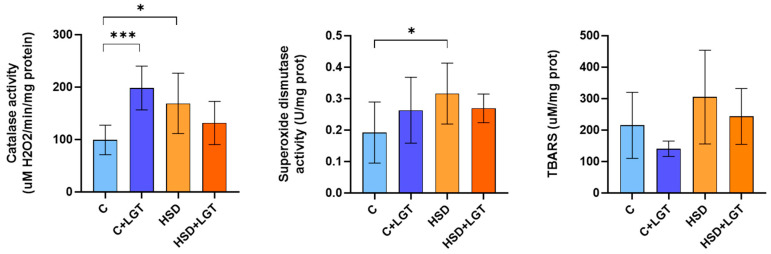
Oxidative stress markers. Catalase and superoxide dismutase activities and TBARS levels in epididymal adipose tissue (EAT) from the Control (C), Control + LGT (C + LGT), HSD, and HSD + LGT groups. Data are presented as means ± SDs. One-way ANOVA followed by Bonferroni’s post hoc test. * *p* < 0.05, *** *p* < 0.001.

In EAT from HSD-fed animals, the activities of catalase (CAT) and superoxide dismutase (SOD) were significantly increased compared to the C group (*p* < 0.01 and *p* < 0.05, respectively). No significant differences were observed between the HSD and HSD + LGT groups. Although TBARS levels were not significantly different, a trend toward higher lipid peroxidation was detected in the HSD group relative to the C group (Figure 6).

### 3.8. Mitochondrial Characteristics

**Figure 7:**
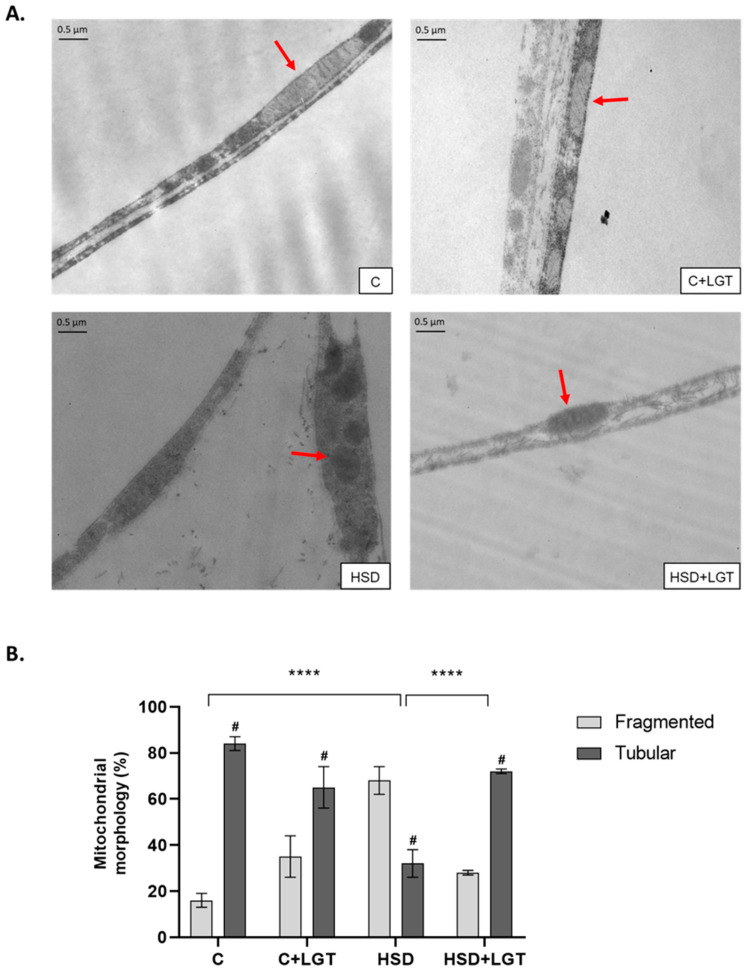
(**A**) Analysis of mitochondrial morphology in adipose tissue by transmission electron microscopy. Representative electron microscopy images of mitochondrial morphology (20.00× magnification). (**B**) Tubular and fragmented mitochondria were counted per arbitrary area and expressed as percentages. Red arrow indicates mitochondria. Two-way ANOVA (factors: mitochondrial morphology and treatment group) followed by Bonferroni’s post hoc test. # *p* < 0.0001 vs. fragmented mitochondrial. **** *p* < 0.0001.

Mitochondrial morphology was evaluated by TEM. In the C and C + LGT groups, mitochondria were predominantly tubular-shaped (84 ± 3 and 65 ± 10%, respectively), while in the HSD group, mitochondria were mostly spherical (67 ± 5%), indicating a prevalence of fragmented mitochondria in comparison with the C group (*p* < 0.0001). As a result of LGT treatment, EAT from the HSD + LGT group presented a significant increase in tubular-shaped mitochondria compared to the HSD group (72 ± 1 vs. 33 ± 5%, respectively, *p* < 0.0001) (Figure 7).

### 3.9. UCP-1 Expression

**Figure 8:**
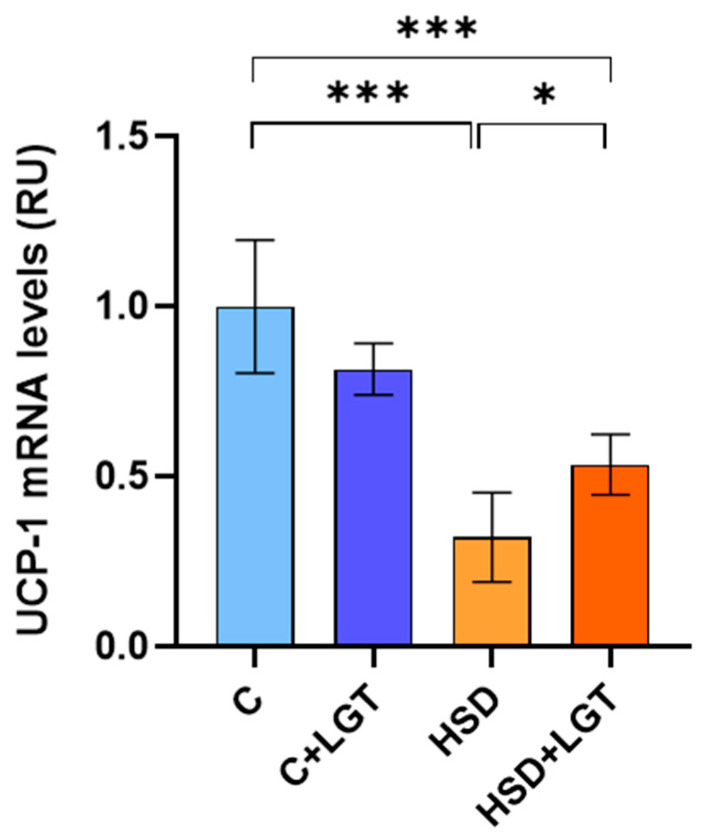
UCP-1 expression. UCP-1 mRNA levels in epididymal adipose tissue (EAT) from the Control from Control (C), Control + LGT (C + LGT), HSD, and HSD + LGT groups. Data are presented as means ± SDs. One-way ANOVA followed by Bonferroni’s post hoc test. * *p* < 0.05; *** *p* < 0.001.

Since UCP-1 is associated with ROS production and mitochondrial function, mRNA levels were quantified by RT-qPCR. UCP-1 expression was significantly reduced in the HSD and HSD + LGT groups compared to the C group (*p* < 0.001). LGT upregulated UCP-1 mRNA levels compared to the HSD group (*p* < 0.05) (Figure 8).

### 3.10. Leptin and Adiponectin Expression

**Figure 9:**
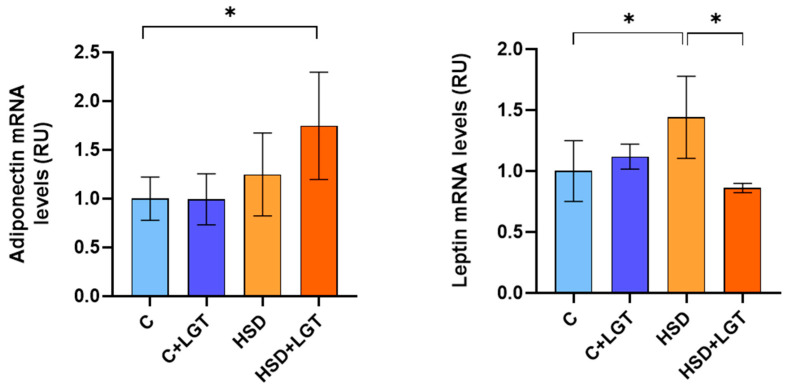
Adiponectin and leptin expression. Adiponectin and leptin mRNA levels in epididymal adipose tissue (EAT) from the Control (C), Control + LGT (C + LGT), HSD, and HSD + LGT groups. Data are presented as means ± SDs. One-way ANOVA followed by Bonferroni’s post hoc test. * *p* < 0.05.

To assess adipocyte function, leptin and adiponectin mRNA levels were measured by RT-qPCR. EAT from HSD-fed animals showed a significant increase in leptin expression compared to the C group (*p* < 0.05), with no changes in adiponectin levels. In contrast, the HSD + LGT group exhibited a significant reduction in leptin expression accompanied by a marked increase in adiponectin levels (Figure 9).

## 4. Discussion

In the present study, we evaluated in an animal model the consequences of chronic salt intake on VAT homeostasis and the beneficial effects of LGT administration. Our study demonstrates that excessive salt consumption induces structural and functional changes in VAT, including reduced adipocyte size and vascular density, increased fibrosis, oxidative stress, mitochondrial fragmentation, and impaired expression of key functional genes. Treatment with LGT partially reversed many of these deleterious changes, supporting its potential role as a modulator of VAT remodeling.

Ultra-processed food intake and the habitual addition of salt is a common dietary pattern in Western countries and is strongly associated with the development of hypertension and CVD [1,2,3]. Despite international guidelines recommending a daily salt intake of less than 5 g [5], average consumption in the general population often exceeds 10 g per day [6,7]. Recent evidence suggests that high salt intake may directly impact tissue metabolism, activating pathophysiological pathways besides its well-established effects on blood pressure [8,19,20].

Considering that dysfunction of VAT is strongly associated with CVD, it is crucial to elucidate the effects of high salt intake on this tissue to gain deeper insights into the mechanisms by which dietary factors contribute to the cardiovascular risk. Studies addressing the impact of short-term salt consumption have reported hyperphagia in animals and humans, accompanied by stable body weight attributed to a hypercatabolic state [11]. Conversely, chronic salt loading has been associated with a higher frequency of insulin resistance, with controversial results regarding its effect on body weight [24], our results show that animals fed an HSD exhibited significant weight loss and a reduced VAT mass, despite an increase in food intake. This reduction could be the result of a high catabolic condition possibly driven by increased sympathetic activation or altered energy partitioning in response to salt loading [11]. Furthermore, in this study, mice fed a long-term HSD exhibited a reduction in the efficiency of food utilization. These findings are consistent with previous studies reporting that HSD intake induces body weight loss and fat mass reduction in rodent models, likely due to a hypercatabolic state [11]. Nevertheless, it has also been demonstrated that diets rich in salt induce changes in the gut microbiota profile [36], which positively correlate with changes in body weight [37]. These mechanisms may at least partially explain the changes in body weight in our model.

Increasing epidemiological, clinical, and experimental data suggest that both AT quantity and alterations in its quality, a condition termed “adiposopathy”, are directly linked to tissue dysfunction. Histologically, the HSD led to a reduced adipocyte volume and increased cell density. It is well established that, under physiological conditions, small, healthy adipocytes are more insulin-sensitive than large adipocytes [38]. However, our study suggests that the reduced adipocyte size observed in the HSD group compared to healthy controls, may represent a pathophysiological feature indicative of impaired VAT remodeling and function.

During VAT remodeling, dynamic changes occur in the extracellular matrix (ECM). The ECM is a complex network of proteins that surround adipocytes, providing structural support and integrating various physiological signals [39]. The plasticity of the ECM is essential for healthy VAT expansion without triggering metabolic dysfunction. However, alterations in the ECM composition or architecture can adversely affect VAT behavior. Excessive interstitial collagen deposition leads to tissue fibrosis and reduces ECM flexibility, contributing to adipocyte dysfunction [39]. Fibrosis generally results from chronic exposure to a wide range of stimuli, such as infections, autoimmune reactions, hypertension, radiation, and tissue injury. Recent evidence suggests that high salt intake activates immune responses associated with the local accumulation of sodium, and promotes the secretion of cytokines, growth factors, and components of the renin–angiotensin–aldosterone system, all of which are involved in profibrotic signaling pathways [40,41]. In our study, the HSD led to a significant increase in the interstitial collagen content in VAT, consistent with the development of tissue fibrosis. Moreover, in our model, fibrosis was associated with a reduced vascular density in EAT, suggesting impaired oxygen and nutrient delivery to adipocytes. In humans, salt loading has been shown to reduce oxygen availability in AT, contributing to local hypoxia [42]. Tissue hypoxia typically induces the expression of HIF-1α to activate the angiogenic response; however, several studies have reported that expression of this factor was higher at the early phase of hypoxia and decreased as hypoxia continued [43]. Therefore, under conditions of chronic oxidative stress, HIF-1α expression can be suppressed due to redox imbalance, impaired transcriptional regulation, or post-transcriptional modifications [44,45]. In an insulin resistance model, we previously demonstrated that VAT expansion was accompanied by a reduced vascular density and decreased HIF-1α mRNA levels, whereas treatment with LGT improved vascularization without affecting HIF-1α expression [46]. In accordance with these findings, our results show that chronic salt consumption also induces a decrease in HIF-1α levels. Significantly, treatment with LGT reduced fibrosis and enhanced vascular density and VEGF expression without modifying HIF-1α expression. These data support the notion that LGT promotes angiogenic signaling and tissue remodeling, probably through mechanisms independent of HIF-1α, potentially contributing to the maintenance of oxygen homeostasis and metabolic function in expanded VAT; however, this hypothesis requires further confirmation. A limitation of the present study is the absence of VEGF downstream signaling and HIF-1α protein analyses, which would have provided additional mechanistic insights into angiogenic pathway dysregulation and its relationship with fibrosis. Based on our results, further research on VEGF downstream signaling is warranted to elucidate the pathways associated with salt consumption. It is also important to note that in our study, HIF-1α mRNA levels were evaluated, whereas the measurement of HIF-1α protein levels would be more representative of its functional activity, as HIF-1α is predominantly regulated and stabilized post-translationally. Future studies including the quantification of HIF-1α protein levels will be essential to elucidate the link between fibrotic remodeling, angiogenesis, and hypoxia under salt overload conditions.

Numerous evidence suggest a link between ECM remodeling and mitochondrial function [47]. Several studies have shown that changes in the ECM composition or excessive matrix deposition can influence mitochondrial dynamics, including processes such as fission, fusion, and bioenergetic activity [47,48]. In this context, mitochondrial function plays a central role in maintaining adipocyte health. Although white adipocytes contain fewer mitochondria than brown or beige fat depots, emerging evidence emphasizes their crucial contribution to energy homeostasis, redox balance, and lipid handling [49]. Mitochondrial dynamics are tightly regulated by the balance between fusion and fission processes. Fusion generates elongated, tubular mitochondria that support oxidative metabolism, while fission produces fragmented organelles that are often associated with dysfunction and impaired energy production. Disruption of this balance has been implicated in various metabolic disorders [48]. In our study, animals fed an HSD showed a prevalence of fragmented mitochondria, suggesting excessive fission and mitochondrial stress, which likely contribute to an impaired bioenergetic capacity [13]. LGT treatment, however, increased the proportion of tubular mitochondria, indicating a restoration of fusion dynamics and mitochondrial integrity. This data is in accordance with our previous study [18] that demonstrates the beneficial effect of LGT on the regulation of mitochondrial dynamics in VAT.

Disruptions in mitochondrial function contribute to oxidative stress generation, which in turn promotes cellular damage [50]. Several evidence shows that excessive salt intake produces an increase in oxidative stress [51]. It is well known that a correlation exists between the mitochondrial membrane potential and ROS production [52]. Under physiological conditions, a small proportion of electrons escape from the mitochondrial electron transport chain and react with molecular oxygen to produce ROS; this process is significantly enhanced when the mitochondrial inner membrane potential is elevated. Uncoupling proteins (UCPs), particularly UCP-1, are key regulators of the mitochondrial membrane potential by allowing controlled proton leakage, thereby preventing ROS overproduction and maintaining redox homeostasis [53]. We have previously reported that both high-sucrose [46] and high-fat diets [18] alter mitochondrial morphology and increase oxidative stress in VAT, accompanied by reduced UCP-1 expression. Consistently, our current findings demonstrate that an HSD increases lipid peroxidation, as indicated by elevated TBARS levels, and suppresses UCP-1 expression. LGT treatment partially restored UCP-1 expression and was associated with reduced oxidative stress markers, suggesting improved mitochondrial function. This partial recovery may indicate a shift toward a beige adipocyte phenotype, which is characterized by enhanced mitochondrial activity, increased energy expenditure, and reduced inflammatory signaling [54].

Changes in VAT induced by high salt intake may be associated with alterations in its functional profile. To assess VAT functionality, we analyzed the expression levels of the adipokines leptin and adiponectin. Leptin levels were elevated in HSD-fed animals, consistent with previous evidence that reveal a link between salt overload, hyperleptinemia, and leptin resistance in rats [51,55]. Notably, LGT reduced leptin expression and increased adiponectin expression, supporting a shift toward an anti-inflammatory adipokine profile. Adiponectin plays a critical role in maintaining VAT homeostasis by enhancing insulin sensitivity, exerting antifibrotic effects, and supporting mitochondrial integrity [56]. Our results support the idea that LGT contributes to restoring AT homeostasis, modulating inflammatory signaling, and preserving cellular metabolism and structural integrity.

Finally, as expected, salt consumption increased SBP and urinary sodium excretion. Additionally, the standardized CrCl was elevated in the HSD group, potentially indicating glomerular hyperfiltration preceding kidney damage. Interestingly, LGT treatment normalized SBP and standardized CrCl, maintaining elevated sodium excretion. These findings suggest that GLP-1 agonists should be further investigated as potential therapeutic agents for hypertension and renal protection.

In VAT, excessive salt consumption adversely affects the ECM composition, vascularization, mitochondrial dynamics, oxidative stress, and adipokine expression. The development of a rigid collagen network within the ECM impairs healthy adipocyte expansion, leading to hypotrophic and dysfunctional cells. LGT treatment ameliorated several of these alterations, suggesting its potential as a therapeutic strategy to counteract VAT dysfunction induced by high salt intake. Further research is needed to elucidate the specific impact of chronic high salt intake on VAT remodeling and to evaluate the clinical applicability of GLP-1RAs as potential modulators of ATdysfunction.

## 5. Conclusions

Our findings provide evidence that chronic high salt intake disrupts VAT homeostasis, even in the absence of obesity or changes in caloric intake. Emerging evidence suggests that VAT alterations, independent of obesity, can contribute to cardiometabolic disorders, including insulin resistance, low-grade inflammation, and endothelial dysfunction [42]. This highlights new potential mechanisms by which excessive salt consumption may increase the cardiovascular risk in non-obese individuals, a population traditionally considered at lower risk. Understanding the early remodeling events in VAT under salt overload could provide new insights into preventive strategies targeting dietary salt reduction, particularly in non-obese individuals.

## Figures and Tables

**Table 1:** General characteristics of the animal model before liraglutide treatment.

	C	HSD
Initial body weight (g)	24.5 ± 1.0	24.3 ± 1.3
Pre-treatment weight (g)	31.3 ± 1.6	25.8 ± 1.6 ***
Food consumption (g/day/100 g b.w.)	19.5 ± 1.7	23.2 ± 3.2 **
Water intake (mL/day/100 g b.w.)	27 ± 5	122 ± 16 ***
Caloric intake (kcal/day/100 g b.w.)	37 ± 4	40 ± 6
Initial SBP (mmHg)	110 ± 9	108 ± 5
Pre-treatment SBP(mmHg)	107 ± 6	125 ± 2 ***

Data are expressed as means ± SDs. HSD, high-salt diet; b.w., body weight; SBP, systolic blood pressure. Unpaired Student’s T test. ** *p* < 0.01, *** *p* < 0.001.

**Table 2:** General characteristics of the animal model and tissue weight at the end of the experiment.

	C	C + LGT	HSD	HSD + LGT
Final body weight (g)	32.5 ± 1.8	31.6 ± 2.1	27.0 ± 2.7 ***^a,^**^b^	26.8 ± 1.7 ***^a,b^
Food consumption (g/day/100 g b.w.)	17.7 (12–29.8)	17.2 (9.6–24.4)	21.8 (16.5–28.2)	19.5 (17.1–23.0)
Water intake (mL/day/100 g b.w.)	24 (19–36)	18 (12–35)	129 (91–158) ***^a,b^	81 (73–108) *^a,b^
Caloric intake (kcal/day/100 g b.w.)	36 ± 8	33 ± 6	38 ± 6	35 ± 3
FER (%)	0.85 (0.59–1.23)	1.05 (0.93–1.05)	0.24 (0.01–0.72) *^a,b^	0.25 (0.20–0.41) *^a,b^
SBP(mmHg)	114 ± 8	103 ± 9	133 ± 10 **^a,^***^b^	117 ± 8 *^b,^*^c^
EAT mass (mg)	614 ± 171	445 ± 158 *^a^	305 ± 100 ***^a,^*^b^	281 ± 67 ***^a,^*^b^
Tibia length (mm)	16.6 ± 0.6	16.4 ± 0.6	16.5 ± 0.4	16.7 ± 0.8
Final body weight/tibia length (g/mm)	2.0 ± 0.1	1.9 ± 0.1	1.6 ± 0.2 ***^a,b^	1.6 ± 0.1 ***^a,b^
EAT mass/tibia length (mg/mm)	36.9 ± 10.0	27.1 ± 8.8	19.0 ± 5.6 ***^a^	17.8 ± 5.1 ***^a,^*^b^

Data are expressed as means ± SDs or medians (ranges) according to the data distribution. LGT, liraglutide; C, Control; HSD, high-salt diet; FER, food efficiency ratio; SBP, systolic blood pressure; EAT, epididymal adipose tissue; b.w., body weight. One-way ANOVA or Kruskal–Wallis test followed by Bonferroni’s or Dunn’s post hoc test according to the group number and data distribution. * *p* < 0.05; ** *p* < 0.01; *** *p* < 0.001. ^a^ vs. the Control group; ^b^ vs. the Control + LGT group; ^c^ vs. the HSD group.

**Table 3:** Circulating biochemical parameters of the Control and high-salt diet groups with and without liraglutide treatment.

	C	C + LGT	HSD	HSD + LGT
Sodium (mmol/L)	151 ± 1	152 ± 2	153 ± 6	153 ± 6
Creatinine (mg/dL)	0.17 ± 0.04	0.13 ± 0.03	0.14 ± 0.03	0.16 ± 0.08
Glucose (mg/dL)	222 ± 34	203 ± 44	229 ± 75	257 ± 75
Triglycerides (mg/dL)	63 (38–91)	67 (44–103)	48 (33–70)	59 (28–85)
Total cholesterol (mg/dL)	92 ± 10	80 ± 11	85 ± 11	89 ± 10
HDL cholesterol (mg/dL)	77 ± 11	66 ± 11	69 ± 13	74 ± 5
Non-HDL cholesterol (mg/dL)	18 ± 6	17 ± 5	17 ± 3	19 ± 3

Data are expressed as means ± SDs or medians (ranges) according to the data distribution. C, Control; LGT, liraglutide; HSD, high-salt diet.

**Table 4:** Urinary biochemical parameters of the Control and high-salt diet groups with and without liraglutide treatment.

	C	C + LGT	HSD	HSD + LGT
Urinary volume (mL/24 h)	0.4 ± 0.1	0.6 ± 0.3	14.5 ± 0.9 ***^a,b^	10.7 ± 2.2 *^a^
uNa (μg/24 h)	47 ± 23	54 ± 24	3186 ± 1046 ***^a,b^	2152 ± 344 *^a,b^
FENa (%)	0.32 ± 0.18	0.27 ± 0.12	8.39 ± 2.56 ***^a,b^	10.70 ± 2.89 ***^a,b^
Creatinine (μg/24 h)	138 ± 38	190 ± 108	287 ± 70	411 ± 233 *^a^
Standardized CrCl (µL/min)	70 ± 30	81 ± 36	150 ± 33 *^a,b^	86 ± 25 *^c^

Data are expressed as means ± SDs. C, Control; LGT, liraglutide; HSD, high-salt diet; uNa, urinary sodium; FENa, excreted sodium fraction; CrCl, creatinine clearance. One-way ANOVA followed by Bonferroni’s post hoc test. * *p* < 0.05; *** *p* < 0.001. ^a^ vs. the C group; ^b^ vs. the C + LGT group; ^c^ vs. the HSD group.
